# X-Ray Treatment as a Preventive of Recurrence Following Extirpation of Malignant Growths*Read before the North Carolina Medical Society, Charlotte, N. C.

**Published:** 1906-10

**Authors:** W. D. Witherbee

**Affiliations:** Charlotte, N. C.


### Abstracts and Selections

### X-Ray Treatment as a Preventive of Recurrence Following Extirpation of Malignant Growths.*

BY W. D. WITHERBEE, M. D., CHARLOTTE, N. C.

There is probably no one disease in medicine which is of such general interest to the medical fraternity as this; not alone to the specialist, but to every general practitioner, for there is not an organ or tissue in the body that may not under the proper circumstances be invaded by some form of malignant growth.

For convenience of description it might be well to divide all malignant growths into superficial and deep. The superficial being those forms that are ordinarily called epitheliomata. The deep including the various forms of carcinoma and sarcoma.

The most chronic and least malignant form of epitheliomata is the rodent ulcer, which usually makes its appearance late in life, somewhere about the eye or nose. It is usually first noticed by the patient as a small scab or crust, which soon drops off, only to form again, each time the ulcer itself gradually increasing in size and depth. This form, therefore, continues in its growth only by continuity and contiguity of tissue, in which case the line of travel of the cancer is infiltrated by cancer cells, invading only the tissues in the immediate neighborhood and not being carried to the glands and various organs of the body by either the blood or lymphatic vessels. Its growth is slow but progressive and will, if not checked, destroy the life of the patient.

---

*Read before the North Carolina Medical Society, Charlotte, N. C.

The other forms of epitheliomata often start in the various warts and moles, which for some unknown reason generally attributed to chronic irritation, suddenly take on a malignant process, which varies much in degree of malignancy; or it may start in an old ulcer as in the case of epitheliomata of the tongue. This form may have secondary deposits in the various lymphatic glands that filter the lymph coming from the part affected. This lymphatic involvement from any tumor or ulcer depends largely on the rapidity of its growth.

The treatment recommended in these cases is the use of a paste which may contain any of the deep caustics or escharotics, or treated surgically by complete removal, or by X-ray.

In some of the more chronic and least malignant cases the paste will sometimes remove the condition; however, this, too often fails, and only sets up an irritation that produces a great activity of cell proliferation and thus lights up a most rapid malignant condition so that the use of another plaster may be practically impossible. In removing cancer we are told by the surgeon to cut wide of the disease; this done, it often happens that the disease soon after returns in the same place. Even when specimens are taken from the wound after the tumor has been removed and pronounced normal by the pathologist the disease may return in the scar. This goes to show that the infiltration of normal tissue by cells that may be carried from the tumor and have not yet reached the stage of development which would indicate their character so that one can not absolutely, even with a microscope, distinguish between a normal cell and a pathological cell.

If the X-ray is used in these cells, especially at the time when most cases are treated with caustics, at least 98 per cent of them will disappear completely. When exposing the patient to the ray it is essential that the part exposed should extend well out into the normal tissue as well as the diseased area.

The subcutaneous and deep malignant conditions invariably in the course of time spread either to the lymphatic glands or as in the case of melonotic sarcoma, which is spread to every organ in the body by the blood stream. So that on post-mortem examination of a case of melono sarcoma one finds metastaces in almost every organ and tissue of the body.

Among the subcutaneous variety the ordinary schirrus carcinoma of the breast will furnish the best example. In these cases the first signs of the disease is marked by the patient noticing a small tumor in the lower and outer quadrant which may be discovered accidentally or her attention being called to it by a few sharp pains in this region. The patient is then referred to a surgeon, who operates immediately. The operation consisting of a thorough and complete removal of the breast axillary and clavicular glands and usually a careful dissection of the deep fascia and a portion of the pectoral muscles. Even with those cases where this most extensive dissection is carried out there are many recurrences.

In doing this operation the best authorities advisé first dissecting out the glands and finally removing the breast, taking out if possible the glands, breast, etc., in one mass. The reason given for this procedure is that it has become an established fact that the local recurrence of the disease may be due to infecting the wound with some of the cancer cells during the operation.

From the reports of the recent cancer search committees it looks at present as though there is no definite germ of cancer and that the exact cause or origin is as yet undetermined, also that a cancer cell when carried from the original site has the power to grow and produce a malignant condition in another organ or tissue.

That the glands in the axilla are involved in schirrus carcinoma of the breast is well known, although they may not show any evidence of it for some time. If the condition is not of a schirrus variety, but of more rapid growth and hence softer and more fungating, the glands are involved much earlier. This, therefore, indicates that the more rapid growth and the softer the tumor the more quickly are the cells liberated from the mass and carried by the lymphatics to the neighboring glands.

The process of invasion of both the local tissue and the lymphatic glands might be compared for the sake of illustration to a sponge filled with water, surrounded by a layer of cotton placed in a rubber bag from which various small tubes may extend. Any pressure then on this bag will express the water from the sponge to the cotton and then out through the various tubes. The sponge representing the tumor, the cotton the local tissue and the tubes the lymphatics.

Now, as these rapidly growing cancers, as well as the schirrus variety, are often found in regions where it is practically impossible to avoid pressure, it is therefore true that malignant conditions occurring in these regions spread faster and produce fatal results much sooner than malignant conditions in other locations.

Suppose, then, the surgeon in operating on a case of this kind, has carried out a most complete dissection and removed the whole mass of glands, breast and tissue, as recommended, and has kept wide of the disease, only cutting in healthy tissue, and then has a recurrence.

I believe that the operation at best is crude for this reason. The object of the surgeon in these cases is to remove every cancer cell that is present, and this he can not do until some means is devised to point out just how far the cells have been carried into the neighboring tissues and glands, even though he does not infect his wound directly by opening into the original tumor itself and allowing it to discharge some of its cells into the wound.

Some of these cases do not recur, and in those that do recur, some time elapses before it makes itself evident, provided the operation has been complete, therefore I believe that before long all surgeons will consider a course of X-ray treatment immediately after the operation as essential as the operation, in that it will destroy the few remaining cells which cause the recurrence, and in this way greatly increase the percentage of complete cures.

In regard to the time the X-ray treatment should be begun, the patient should be treated on a stretcher the day following the operation. This may seem rather a radical procedure, nevertheless the cells which may be in the open wound or in the adjacent tissue will be more completely destroyed in a much shorter time when the wound is fresh and open than when one waits for union before beginning treatment. The open wound not only greatly facilitates the direct action of the ray on the remaining cells, but also affords free drainage for all the lymphatic vessels in this region which may be laden with cancer cells.

In cases where radical operation is impossible and the chances of complete recovery are almost nil, the course of X-ray treatment will lessen the discharge, control the hemorrhage and to a surprisingly great extent allay the pain so that the patient during these last hours will be made more comfortable, and thus die of exhaustion similar to cases of pernicious anemia and old age, instead of the suffering and agony usually seen in the last stages of this disease.—*Charlotte Medical Journal.*

